# Languedoc lagoon environments and man: Building a modern analogue botanical macroremain database for understanding the role of water and edaphology in sedimentation dynamics of archaeobotanical remains at the Roman port of *Lattara* (Lattes, France)

**DOI:** 10.1371/journal.pone.0234853

**Published:** 2020-06-18

**Authors:** Bigna L. Steiner, Natàlia Alonso, Patrick Grillas, Christophe Jorda, Gaël Piquès, Margaux Tillier, Núria Rovira

**Affiliations:** 1 Department of Environmental Sciences, Integrative Prehistory and Archaeological Science (IPAS), University of Basel, Basel, Switzerland; 2 Archéologie des Sociétés Méditerranéennes (ASM), UMR5140, CNRS, Paul Valéry University of Montpellier, Montpellier, France; 3 Departament d’História, Grup d’Investigaciò Prehistòrica, Universitat de Lleida, Lleida, Catalonia, Spain; 4 Tour du Valat, Research Institute for the Conservation of Mediterranean Wetlands, Arles, France; 5 Inrap-Méditerranée, Parc Actipolis, Villeneuve-lès-Béziers, France; 6 Institut des Sciences de l’Evolution de Montpellier (ISEM), Montpellier, France; University of Liverpool, UNITED KINGDOM

## Abstract

A new method to evaluate archaeological wetland sites in a more objective way was tested. Different wetland environments have been sampled in areas of a nature reserve and their macroremain content analysed to build a modern analogue dataset. This dataset was then used to characterise archaeological samples from a navigation channel from the Roman port city *Lattara*. In the modern analogue samples, the different wetland types (saline/brackish or fresh water) could be differentiated in the correspondence analysis. Within these groups, the sampled area of the littoral (submerged, shoreline, unsubmerged) could also be differentiated. This dataset can therefore provide a basis for the interpretation of the nature and degree of aquatic influence and layer formation processes in archaeobotanical records of coastal sites. In the tested archaeological samples from the navigation channel of *Lattara*, changes in space and time could be tracked using the modern analogue dataset and geoarchaeological information. The channel lost its fresh water supply and silted up over a short period of time (approx. 100 years).

## 1. Introduction

Wetlands have been an important focus of human activity since prehistory due to their wide range of valuable resources, e.g. resources such as food and water, easy transportation on boats, buffering of extreme weather conditions, diversity of habitats etc. [1, 2]. These in many aspects highly dynamic environments could also severely test the resilience of their inhabitants and lead to a complex entanglement of natural and cultural factors [3–5]. But one thing remained constant: the changing water levels would leave traces in the archaeological sediments, which can be used to assess human responses to their changing surroundings. Interpreting and disentangling these traces remains a very difficult task as there is no modern analogue nor experimental data available [6, 7].

The use of modern analogue data sets can provide a more objective tool to interpret archaebotanical data in order to allow the assessment of the specific conditions of past habitats. The most prominent example is the application of the functional attributes of weeds to recognize crop husbandry practices based on archaeobotanical weed assemblages (FIBS method, e.g. [8–10]). In combination with the use of stable isotope analysis, this method has led to informative research in the field of early agriculture [11]. Other approaches comprised the studies of modern assemblages, e.g. regarding cereal processing [12–14] or dung composition [15–17]; for pollen-only see [18, 19], in order to advance archaeobotanical interpretations. [20] compared data from vegetation surveys with Neolithic drift litter while [21] compared plant remains to existing plant communities of the Doubs river. For micro-remains (pollen and phytoliths), studies of modern analogue data were generally more often integrated into archaeological interpretations than for macro-remains [22–26]. [27] compared modern analogue phytolith assemblages of different types of rice fields and carried out modern ethnographic studies in order to inform the interpretation of archaeological phytolith assemblages [28].

Modern analogue data on seed banks, standing vegetation or a combination thereof have previously been compared with fossil (but non-anthropogenic) assemblages [29–33]. Modern analogue seed bank studies from wetland environments were also used for the interpretation of archaeobotanical data of Roman and medieval samples [34] as well as Neolithic lakeshore sites [35, 36, based on a dataset by 37]. Aside from these limited applications, there is a lack of integration of archaeobotanical and modern analogue seed bank data [38] despite the fact that several studies on seed banks from various habitats are readily available (e.g. in aquatic and wetland habitats: [39–49]). [38] has stressed the similarities in main research questions between the fields of archaeobotany and ecology when it comes to aiming to link standing vegetation with associated seed deposition and survival in the soil. In both cases, several taphonomic issues complicate the link from seed bank to standing vegetation (e.g. variability of seed production, selective mode of preservation, post-depositional disturbances). By directly comparing the modern analogue seed bank to the archaeological, instead of taking a detour via the standing vegetation, several of these issues can be eliminated to a certain degree (e.g. differences in seed production, persistence of seed banks, preservation of seeds, their dispersal, seed transport and the resulting lack of phytosociological relation occur in both modern analogue and archaeological seed banks; [38, 40, 50, 51]). Of course, the influence of human activity on the natural environment represented in archaeological samples cannot be clearly evaluated [38]. By looking mainly at what is considered to have been naturally deposited seeds, this influence can be diminished. Other problems can arise from this approach, for example anthropogenic changes in the environment (wetlands specifically were strongly affected by humans, e.g. [2]) or the appearance of invasive species in modern analogue samples. The latter can be overcome by the use of ecological groupings instead of individual species as a basis for the evaluation, where less value is attached to unusual species. Some problems remain, such as sampling and methodological differences. It is for this reason that we wanted to test the utility of the strategy of using modern analogue samples in order to interpret archaeological samples in [36] in another geographic region and for another archaeological time period.

**Fig 1:**
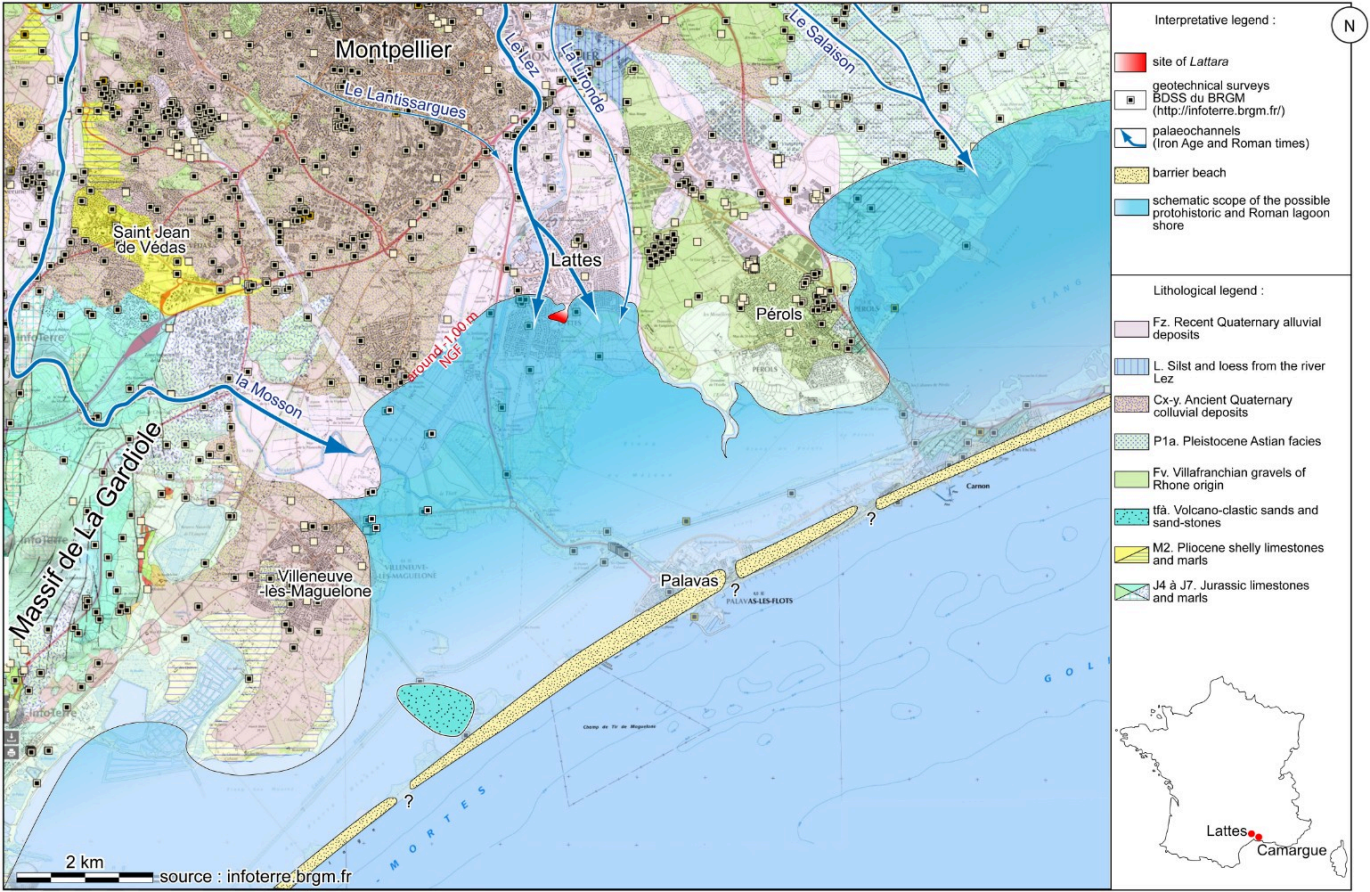
Geomorphological interpretation of the *Lattara* area and coastline during Iron Age and Roman times. The site of *Lattara* is marked in red.

The ancient port city of *Lattara* was considered a suitable site for such a project, as it was situated at the mouth of a river (the river Lez) on the edge of a lagoon [52] and thus influenced by these two sources of water (Fig 1). *Lattara* is a fortified trading post founded around the 5^th^ century BC by indigenous people, as well as Etruscan and Greek merchants, and mainly occupied until the 2^nd^ century AD [53]. The actual archaeological project concerns the port installations situated outside the walls, from which we can highlight a navigation channel and a commercial/artisanal quarter [54–57]. The samples used for this article originate from waterlogged sediments of the channel and are globally dated from the 2^nd^ century BC to the 2^nd^ century AD, even if new ^14^C dates done on one of the surface samples extend the chronology of this zone until the 9^th^ century AD [57].

In the Nature Reserve of the Grand Camargue, comparable modern analogue samples could be found in a protected area that is less anthropogenically influenced than elsewhere. This area is officially protected since 1975, although protection efforts already started in 1927. Today it is a Regional nature park, a Ramsar site, a Natura 2000 site and includes several nature reserves and non-classified protected areas. In Lattes, modern analogue samples originate from wetlands surrounding the archaeological site.

Our main research questions were the following: (1) Can the different tested wetland types in the modern analogue samples be well differentiated from each other? (2) Is it possible to interpret taphonomic and formation processes in archaeological plant assemblages of the navigation channel of *Lattara* based on modern analogue samples? (3) Which wetland types (lagoon, river) having influenced the ecosystems around the navigation channel of *Lattara* can be differentiated in sediment using modern analogue data? (4) How strongly did water influence the sediments of the navigation channel of *Lattara*?

## 2. Material and methods

### 2.1. Modern analogue samples (MAS)

**Fig 2:**
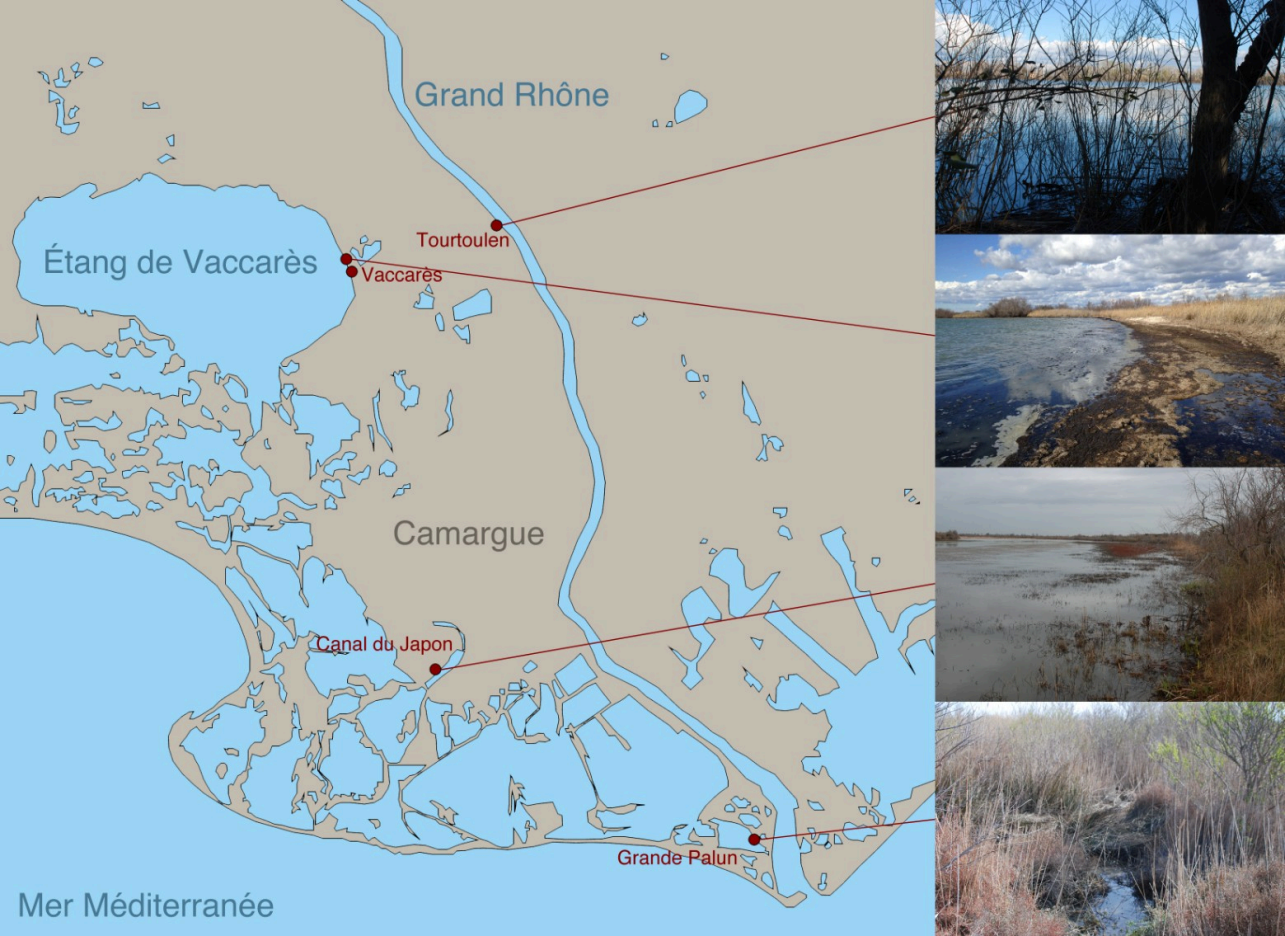
Sampled sites in the Camargue: The riparian forest *Tourtoulen*, the lagoon *Étang de Vaccarès*, the channel *Canal du Japon* and the lagoon *Étang de la Grande Palun*.

**Fig 3:**
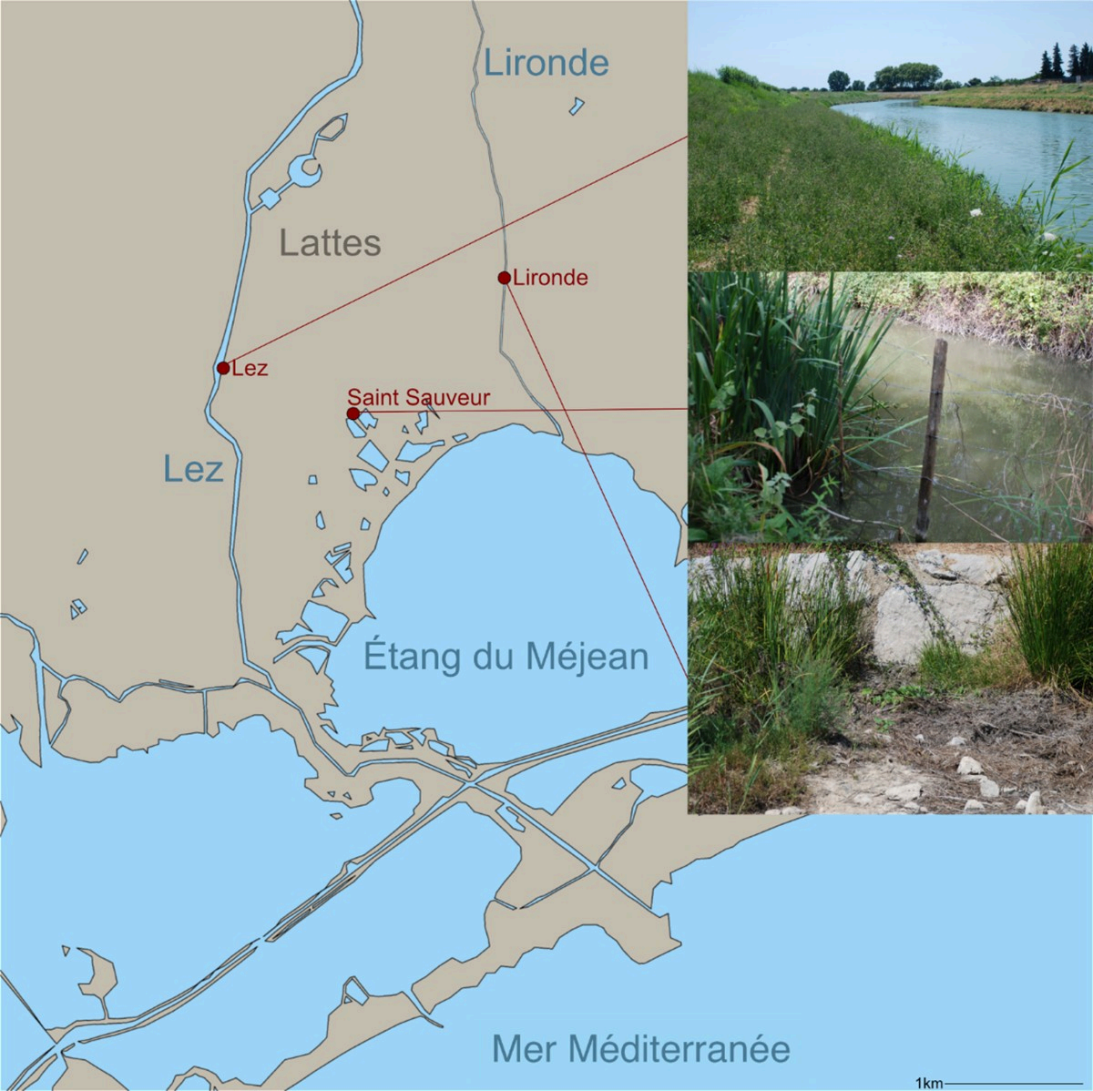
Sampled sites around Lattes: The shores of rivers *Lez* and *Lironde* and two canals in the *Saint Sauveur* area.

Study sites for the modern analogue botanical macroremain database were mainly based in the naturally protected areas of the Grande Camargue in the beginning of March 2018 (Fig 2). The modern analogue samples were taken in transects perpendicular to the shoreline along moisture gradients. The lengths of the transects and number of samples were adapted to conditions at the different sites and roughly followed plant communities on the shoreline. The water salinity as well as the local vegetation were recorded (see S1 Table, sheet ‘sample information’, for salinity, classification into fresh, brackish and saline water, and recorded species). Some additional individual samples were taken in the area of Lattes, at approx. 1.5 km distance to where the archaeological site is located, in the middle of July 2018 (Fig 3).

#### 2.1.1. Étang de Vaccarès

This is a large and shallow lagoon (67km^2^, 1.4m mean water depth; [58, 59]), connected indirectly to the Rhone delta and the Mediterranean sea, also it is strongly influenced by rainfall and wind and therefore has varying levels of salinity [58, 60]. In the last 30 years the salinity fluctuated between 4 and 32 PSU [61]. The lagoon is protected since 1927, but was left untouched for centuries except regarding water management. It receives drainage water from the surrounding land including agricultural fields and the connexion to the sea is managed by sluices.

Two transects were sampled at this lagoon (with permission of the Reserve Naturelle Nationale de Camargue). One transect was close to a man-made fresh water canal, but the water was nonetheless saline during sample extraction. This transect was the longest from this study with 7 samples spread over approximately 250m. The other transect was close to the road and spread over only 35m, but only 3 (two submerged samples and one from the shoreline) out of 5 samples were analysed due to the short duration of this project. During the extraction of the samples, the water at this transect was saline as well. The standing vegetation at both transects primarily comprised: *Sarcocornia fruticosa* (L.) A.J. Scott, *Halimione portulacoides* (L.) Aellen, *Limbarda crithmoides* (L.) Dumort., and *Symphyotrichum squamatum* (Spreng.) G.L. Nesom.

#### 2.1.2. Canal du Japon

Man-made irrigation channel in an ancient river branch of the Rhone (Bras de Fer, active from 1587 to 1711; [62, 63]), activated in 1754 with fresh water from the Rhone [64, 65]. The channel receives drainage water from agriculture. It is the only sampling spot in the Camargue which is not officially protected.

One transect was sampled. The transect spread over approximately 100 metres, including 4 samples, three taken from submerged conditions. During the extraction of the samples, the water of the channel was saline. The standing vegetation primarily comprised: *Sarcocornia fruticosa* (L.) A.J. Scott, *Juncus maritimus* Lam., *Phragmites australis* (Cav.) Steud., *Stuckenia pectinata* (L.) Börner and *Ruppia cf*. *cirrhosa* (Petagna) Grande.

#### 2.1.3. Étang de la grande palun, La palisade

Situated in the estuary of the Rhône (120ha, mean depth: 0.6m; [66]), influenced both by the river and the sea by varying degrees. Since the water is influenced by the Rhone, it is also, to some degree, polluted with high values of dissolved nitrogen mainly related to agricultural activity. Because of shallow depth and wind exposure it is often turbid and therefore, it does not contain many aquatic species (e.g. only two species were found at nine sampling spots in 2010; for full species richness and density data see [66, p. 39, 67].

One transect was sampled here, spreading over approximately 40 metres, including 5 samples (with permission of the Parc Naturel Régional de Camargue). During the extraction of the samples, the water of this lagoon was brackish. The standing vegetation primarily comprised: *Phragmites australis* (Cav.) Steud., *Tamarix gallica* L., *Sarcocornia fruticosa* (L.) A.J. Scott and the invasive species *Baccharis halimifolia* L.

#### 2.1.4. Tourtoulen

Riparian forest (44ha) on the right bank of the Grand Rhone which has been left unused since the late 60s/early 70s (after the last wood harvest) and protected since 1987. The forest is located between the river and the dyke and is thus fully exposed to fluvial influence. It is still occasionally inundated [68], but the succession is ongoing: *Populetum albae* among other communities [69, also 70, 71]. Samples were taken in the southern sector, where there is less erosion than in the northern sector (the northern sector is located at the exit of the meander of the Grand Rhone, while this current is deflected in the southern part, see [72]).

The transect in this forest spread over approximately 200m, including 5 samples. During the extraction of the samples, the water of the Rhone was confirmed to be fresh. The standing vegetation primarily comprised: *Populus alba* L., *Fraxinus angustifolia* Vahl, *Quercus ilex* L. and *Q*. *pubescens* Willd., *Hedera helix* L., *Rubus sp*. L., *Laurus nobilis* L. (which is native but showed a strong population increase within the community; [71]), and the invasive species *Amorpha fruticosa* L.

#### 2.1.5. Lattes

Two samples were taken from the River Lez in the south of Lattes. Currently, this short Mediterranean coastal river crosses Lattes before flowing into the sea. The site of *Lattara* used to be situated in its delta [52], but due to ongoing progressive siltation, the delta is now further south. The salinity was not measured at this location, but was assumed to be fresh (like all river water, see e.g. [73]). The standing vegetation primarily comprised: *Aristolochia clematitis* L., *Convolvulus sepium* L., *Urtica dioica* L. and the invasive species *Ludwigia peploides* (Kunth) P.H. Raven and *L*. *grandiflora* (Michx.) Greuter & Burdet.

Two samples were taken from the short river Lironde, situated at the east of Lattes and flowing into the Méjean lagoon, as the river Lez did during the Roman period. This stream was completely dried out during sample extraction in July 2018, although it is likely that the dryness was only temporary. The standing vegetation primarily comprised *Alisma* cf. *lanceolatum* With., *Typha* sp. L., *Beta vulgaris* L., and *Lythrum salicaria* L.

Two samples were taken from two canals near the Saint Sauveur area just south of the site of *Lattara* and north of the protected area of the Méjean lagoon (Fig 3). One of the channels was also completely dried out during sample extraction and it is unclear when it was last submerged. The water in the other channel was fresh during the extraction of the samples. The standing vegetation primarily comprised: *Iris pseudacorus* L., *Lythrum salicaria* L., *Fraxinus angustifolia* Vahl, *Verbena officinalis* L., *Althaea officinalis* L.

### 2.2. Archaeological samples

As mentioned in the introduction, the archaeological samples originated from the Gallo-Roman port city of *Lattara*, an important commercial enclave during Antiquity, dated from the 5^th^ century BC to the 2^nd^ century AD [53, 74, 75], even if current archaeological work shows the survival of activities in the port area at least until the 9^th^ century AD [57]. This site was situated at the mouth of the River Lez on the edge of the lagoon “*stagnum latera*” mentioned by Pliny the Elder’s Natural History [52]. During the ancient occupation of the site, the Lez embraced the site with two channels (Lez occidental and oriental) forming a lobate delta, leading to the site’s position as a peninsula within the lagoon [52, 76] (Fig 1), but during the Roman times the port area, situated outside of the southern city walls, was already partially silted up and new installations were built [57].

The site is being excavated and interdisciplinarily investigated over thirty years, leading to a rich pool of data from a multitude of different fields: archaeology, palynology, archaeobotany, archaeozoology, anthracology, geoarchaeology, stable isotopes (e.g. [74] and articles therein; [52, 76–87]). The past natural surroundings evidenced in the above mentioned studies include oak and riparian forests, close-canopy forests, as well as fresh, brackish and saline water environments. Open spaces like meadows and pastures, ruderal and disturbed areas as well as cultivated land are also well-documented (see references mentioned above).

**Fig 4:**
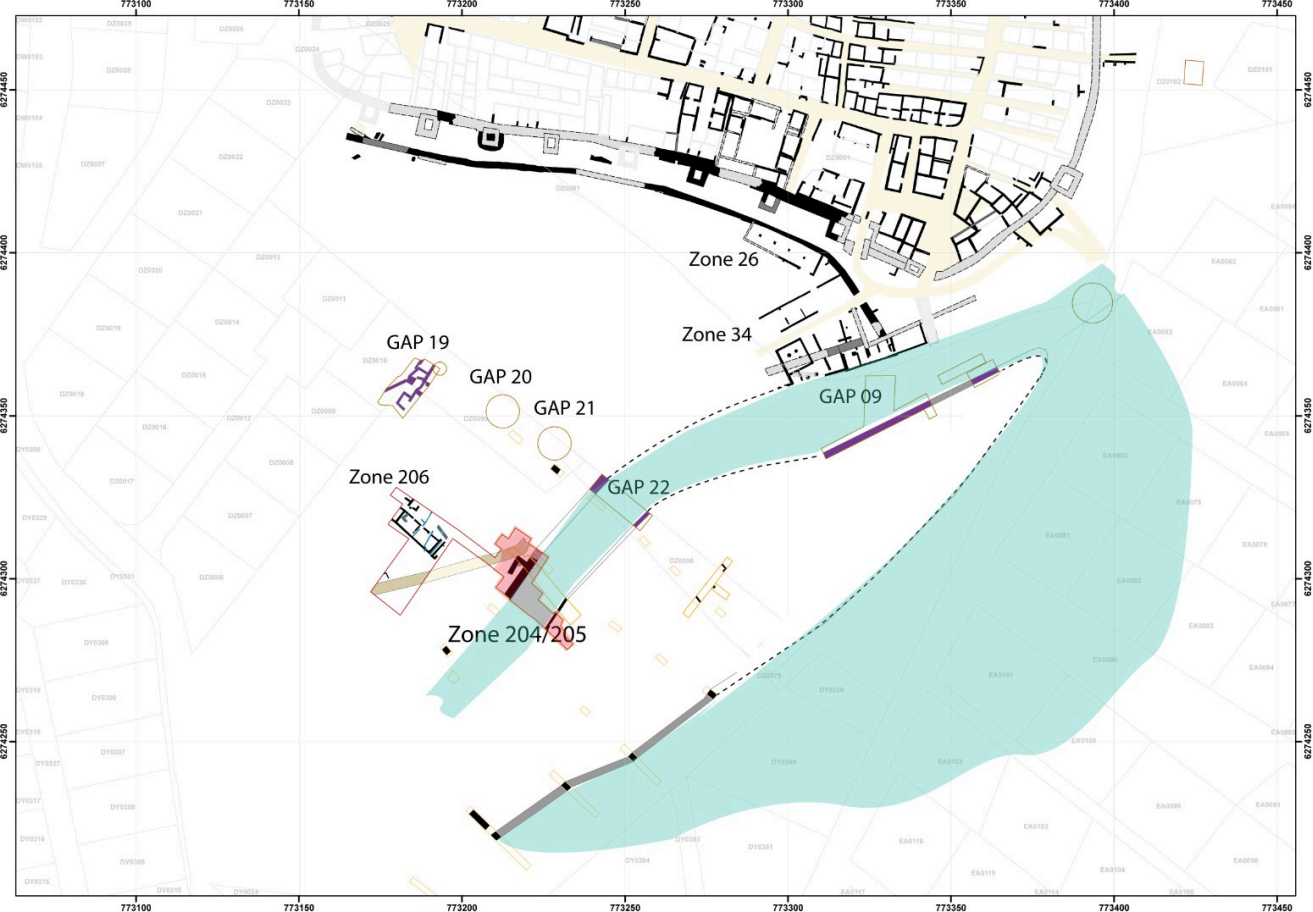
Position of zones 204 and 205 at *Lattara*, where the archaeological samples used in the comparison were taken.

**Fig 5:**
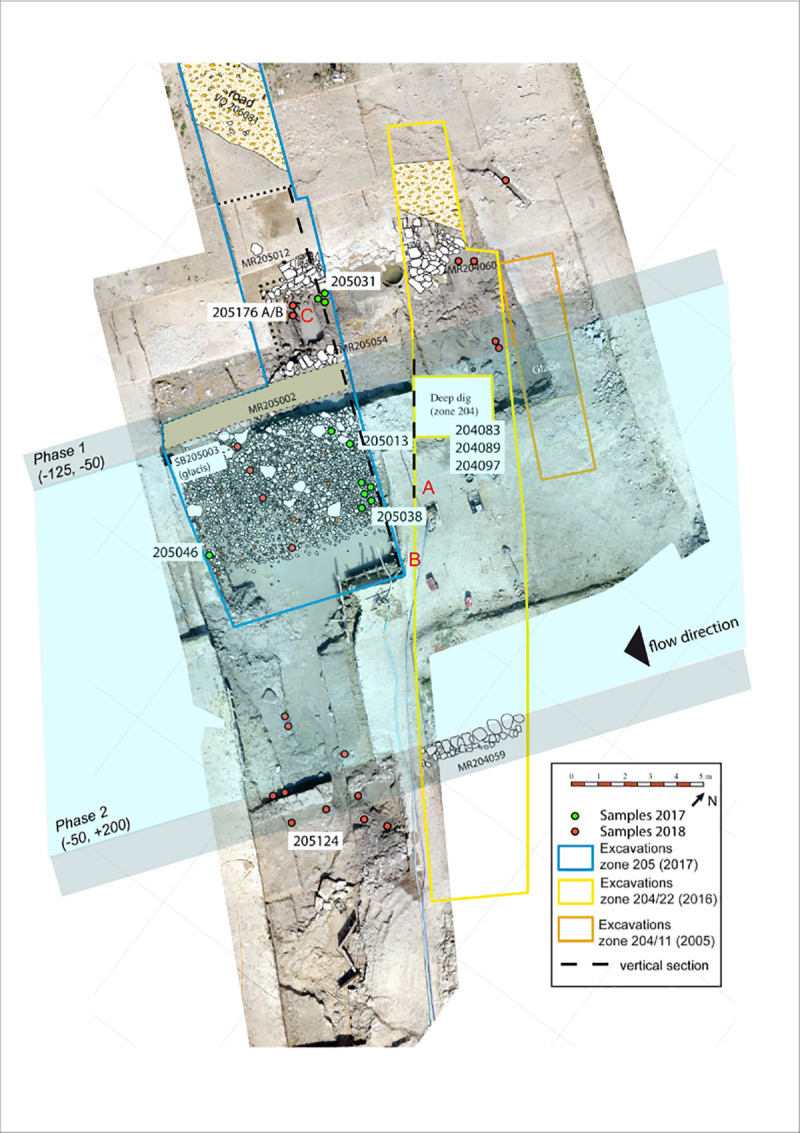
Archaeological samples that were used for the comparison with MAS. The 10 samples are indicated by their sample numbers.

**Fig 6:**
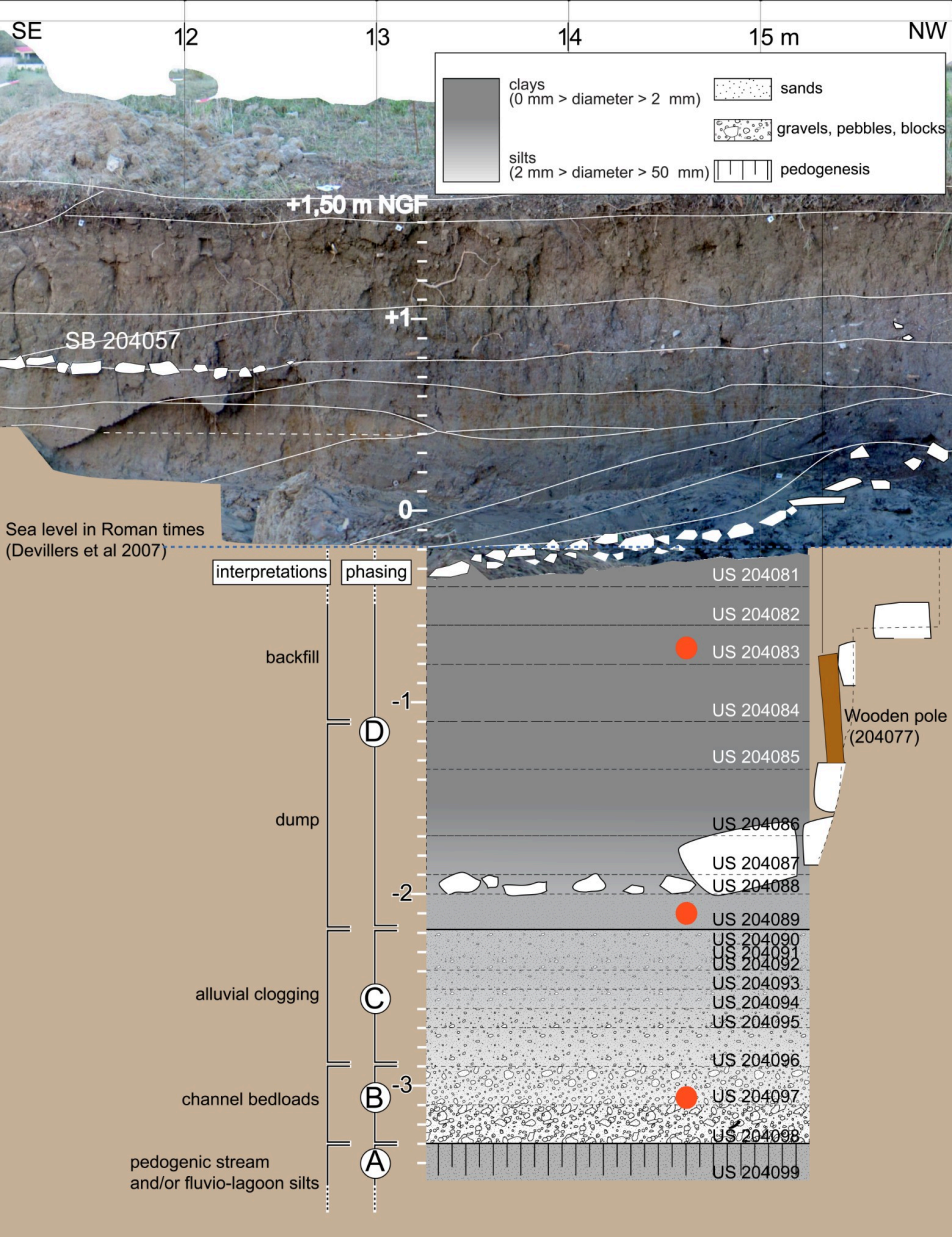
Stratigraphy of the samples that were studied in 2016. (M. Tillier) in zone 204 (in red) and compared here with MAS.

**Fig 7:**
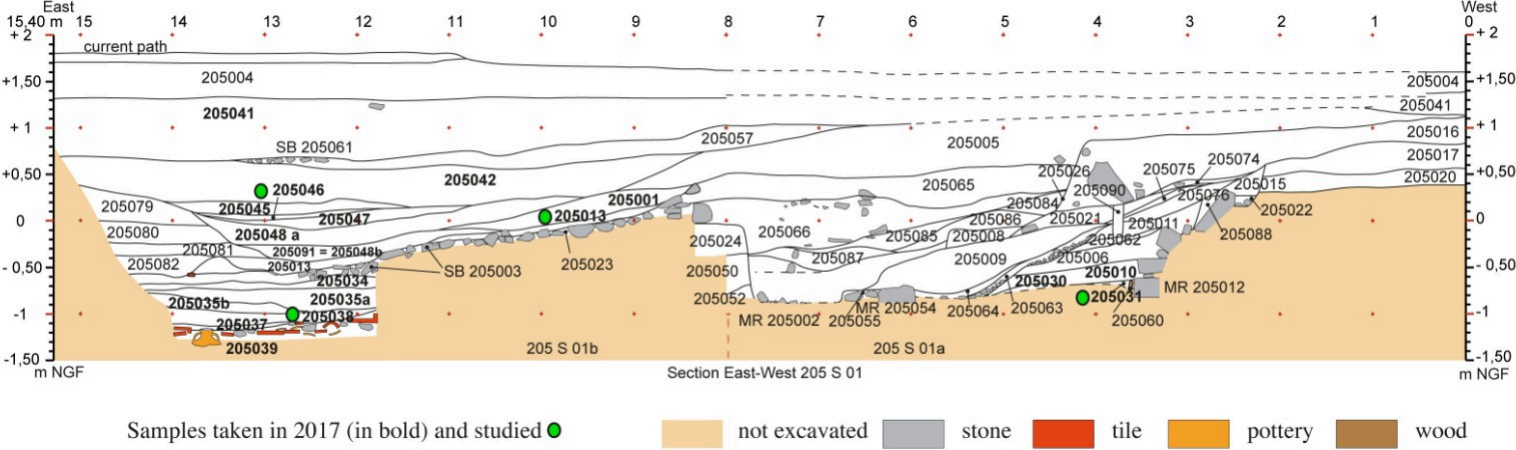
Stratigraphy of the samples that were taken (in bold) and studied (in green) in 2017 (É. Delbois, N. Rovira) in zone 205 and compared here with MAS.

**Fig 8:**
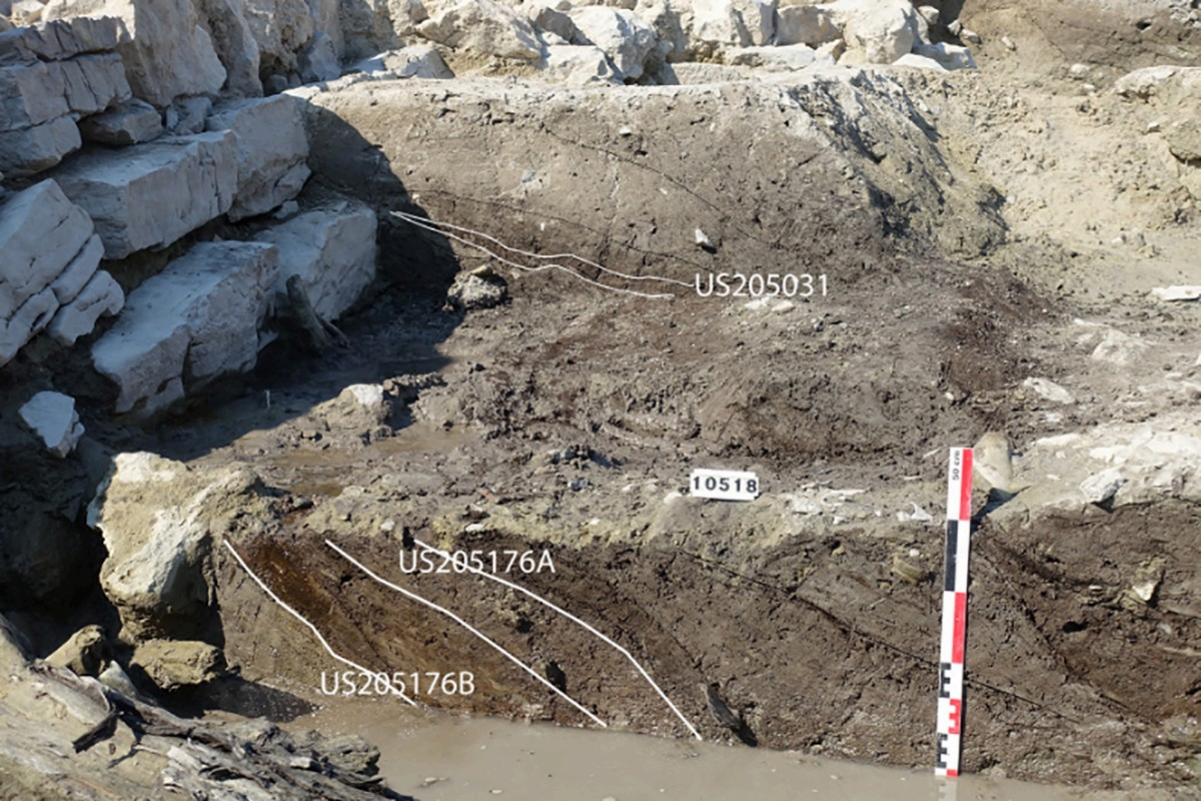
Stratigraphy of two samples that were studied on 2018. (B. Steiner) in zone 205 (US 205176A and B) and compared here with MAS.

For this article, a very restricted number of archaeological samples from this site were used. Samples were only included if they contained waterlogged material and were not dried out before analysis, resulting in only 10 samples (out of >200) as a basis of comparison in this study. These 10 samples all originated from a structure interpreted as a navigation channel at the southwest of the site on the outskirts of the city and dated to the Roman period [54–57] (Fig 4). These samples mainly contained anthropic waste. However, it can be assumed that the plant material in these samples originated at least partly from natural sources. The samples originated from three positions (Fig 4 zones 204, 205, Fig 5), spanning four continuous centuries of occupation. In the zone 204, samples were taken from a survey, done with an excavator in 2016, to reach the lowest layers of the channel [88, 89] (Fig 6; 204097–1 (1/50), 204089–1 (100/150), 204083–1 (150/200)). In the zone 205, samples from two positions were tested. At one position, three surface samples and one profile sample from differently dated layers were analysed in 2017 [90] (Fig 7; 205031–2 (-175/-100; ^14^C 259/108 cal BC), 205038–1 (150/200), 205013–1 (175/225), 205046–1 (^14^C 771/903 cal AD, median probability 849)). At the other position, three surface samples from the borders of the channel and slightly older, were analysed in 2018 [91] (Fig 8; they were chosen to represent the two sides of the channel; 205124–1 (-125; ^14^C -169/3 cal BC/AD, median probability -79), 205176A and B (-175, -100)).

### 2.3. Methods

#### 2.3.1. Modern analogue samples (MAS)

A sampling tube of 25cm diameter was used to sample the uppermost 5cm of the soil, resulting in samples of roughly 2 litres. Samples in the Camargue were collected at the beginning of March 2018, before germination. Additional samples around Lattes were taken in July 2018 to represent a greater diversity of freshwater habitats.

The locations where the MAS were taken were classified as being taken from **submerged** (probably representing the sublittoral), **shoreline** (at the transition between water and land) or **unsubmerged conditions** (landwards of the shoreline, probably representing the eulittoral, supralittoral and epilittoral). These classifications reflect the conditions when the samples were taken and might vary a lot during other times (seasonally but also regarding short-time changes due to strong rainfalls etc.).

The samples were sieved using the wash-over method [92, 93, 94], which was done by the same operator. Most samples were treated with a freezing pre-treatment prior to sieving, which helps to gently disintegrate concretions [95]. This was especially helpful as many samples were very loamy. Mesh sizes of 4, 1, 0.5 and 0.25mm were used in order to fit in with the methodology already used for sieving the archaeobotanical samples. Volumes were measured using the displacement volume [96]. Samples were dried after the sieving process, after an initial test that indicated that modern plant remains were not too strongly affected by this process: three test samples were sorted while being kept wet and only dried after being sorted. The sorted material was then dried and checked again to see how the remains had changed during the drying process. Unlike archaeobotanical remains, the modern analogue material was much more robust against the drying process and did not break readily.

For the sorting, a Euromex NexiusZoom stereo-microscope (magnification 6.5-45x) was used. Seed and fruit remains were sorted and quantified fully based on counting units [97]. Various other macroscopic sediment components in the organic fractions (such as leaves, gastropods, bivalves, ostracods etc.) were semi-quantified (based on a simplified scale by [98]). The smaller sieving fractions were usually subsampled (based on the volume and the richness of the fractions).

The identification of plant remains was done using the seed reference collection of the ASM archaeobotany lab of the university of Montpellier, reference material that was collected in the framework of this project, and literature [99, 100]. It was not possible to determine all plant remains to the species level due to missing seed reference material (some plant species cannot be determined by using photos only). If possible, missing reference material was collected in the field, but due to different ripening times of diaspores and the rarity of some occurring plants, this was not feasible for every plant family.

ArboDat [101] was used to record the samples. By using this archaeobotanically widely used software, the modern analogue data can be reused and distributed among colleagues.

#### 2.3.2. Archaeological samples

The samples used for the comparison were taken and analysed over several years (2016–18) and the methodology as well as the persons processing the samples varied. Seven samples were wet-sieved, while three samples were treated with the same sieving methodology as the modern analogue samples, using wash-over sieving (S2 Table, sheet ‘sample information’). Sieving mesh sizes of 4, 1, 0.5mm were used in all cases, and 0.25mm in some cases, though this fraction was only analysed for the three samples treated with wash-over sieving.

The identification of plant remains was done using the seed reference collection of the ASM archaeobotany lab of the university of Montpellier and literature, e.g. [99, 100].

The archaeological information system (AIS) Syslat-Terminal 5 was used to record all the samples from this site. This system directly connects archaeobotanical results to the archaeological features and the results of other disciplines such as palynology, archaeozoology and anthracology [102, 103].

#### 2.3.3. Data analysis

The classification into ecological groupings was done based on various phytosociological and botanical literature [100, 104–108, www.infoflora.ch; www.tela-botanica.org]; for the zonation of the vegetation along shores, see e.g. [109, chapter 9.3] (river) and [110, p.18] (brackish marsh). Characeae were attributed to aquatic plants favouring oligotrophic conditions because a lot of their representative species prefer such conditions [111]. It was not possible to identify the Characeae oospores in more detail within this short project time. The identification of Characeae is quite difficult: a complete oospore reference collection including all occurring species or a detailed key of the region is needed to do it. It was also not possible to identify the Amaranthaceae in more detail as the missing reference material could not be collected until late in the year (most of the species within this family only flower in July or later) and not for all necessary species. Nomenclature follows [108].

Analysis of the data was performed using correspondence analysis (based on density of a species within ecological groupings) in the program PAST 3.16 [112]. Three samples from water sources that were completely dried out during sampling were excluded, but checked as supplementary variables using the AnalyseSHS tool of the Pantheon-Sorbonne University automatically running an R script (http://analyse.univ-paris1.fr/). They were eventually not included as they did not add value to the results (the drying out had affected the samples in an unpredictable way). The analysis was done based on ecological groupings of aquatic and wetland plants, although individual aquatic and wetland plant species were tested separately as well and gave very similar results. Our expectation concerning the correspondence analysis was to get indicative groups of the modern analogue samples that can be used to classify the archaeological samples.

## 3. Results

### 3.1. General results of MAS

Densities of plant remains in general varied substantially between samples and reached a maximum of 7103 remains/litre (hereafter r/l) to a minimum of 12.7 r/l, with an average of 1418.9 r/l per sample and a median of 532.7 r/l (Table 1). This large variation amongst the samples was most likely caused by the different nature of the sampling spots, although no general links could be made between types of habitats and the density of plant remains. The number of taxa varied between 72 and 5, with an average of 19.1 and a median of 15. In most samples, aquatic and wetland plants made up the majority of plant remains, on average 80% (see S1 Table for the classification of the ecological groupings). However, in some samples, this percentage could be much lower: in three of the samples around Lattes and in the most landwards sample of the transect from the riparian forest of Tourtoulen, aquatic and wetland plant remains made up less than a third of all remains. Other samples with low densities of aquatic and wetland plants (but also of other plants remains) could be found in the two samples from submerged conditions from the second transect of the Vaccarès, but all samples contained more than 10r/l of aquatic or wetland plant remains. Samples from the Canal du Japon had, in general, very high densities of aquatic and wetland plants, as did one sample from the Lironde. The ecological grouping of aquatic plants favouring oligotrophic conditions (namely Characeae) reached highest densities on average, riparian woodland and carr plants the lowest (although they reached higher densities in samples from Tourtoulen).

**Table 1:** Densities and percentages of plant remains in the modern analogue samples.

site name	sample nr.	density total (r/l)	density aquatic and wetland plants (r/l)	density other plants (r/l)	% aquatic and wetland plants
Lironde	L_T2_P1	7103.0	6528.0	575.0	91.9
Lironde	L_T2_P2	56.7	42.0	14.7	74.1
Lez	L_T3_P1	101.5	23.0	78.5	22.7
Lez	L_T3_P2	546.0	176.5	369.5	32.3
Saint Sauveur	L_T5_P1	3887.5	3798.0	89.5	97.7
Saint Sauveur	L_T5_P2	350.0	12.5	337.5	3.6
Étang de la Grande Palun	P_T1_P1	2340.5	2340.0	0.5	100.0
Étang de la Grande Palun	P_T1_P2	2492.5	2486.0	6.5	99.7
Étang de la Grande Palun	P_T1_P3	218.7	211.3	7.4	96.6
Étang de la Grande Palun	P_T1_P4	349.5	348.0	1.5	99.6
Étang de la Grande Palun	P_T1_P5	453.9	436.8	17.2	96.2
Canal du Japon	R_T2_P1	5347.6	5221.2	126.5	97.6
Canal du Japon	R_T2_P2	4193.0	3835.7	357.4	91.5
Canal du Japon	R_T2_P3	3229.2	3069.6	159.6	95.1
Canal du Japon	R_T2_P4	2636.8	2356.4	280.5	89.4
Tourtoulen	T_T1_P1	90.6	75.6	15.0	83.4
Tourtoulen	T_T1_P2	775.0	623.5	151.5	80.5
Tourtoulen	T_T1_P3	334.2	278.4	55.8	83.3
Tourtoulen	T_T1_P4	110.3	108.6	1.6	98.5
Tourtoulen	T_T1_P5	128.6	10.8	117.8	8.4
Étang de Vaccarès	V_T1_P3	861.1	854.8	6.3	99.3
Étang de Vaccarès	V_T1_P4	12.7	12.0	0.7	94.7
Étang de Vaccarès	V_T1_P5	17.0	17.0	0.0	100.0
Étang de Vaccarès	V_T2_P1_1	1179.2	592.3	586.9	50.2
Étang de Vaccarès	V_T2_P1_2	519.4	516.7	2.8	99.5
Étang de Vaccarès	V_T2_P2	2550.0	2518.9	31.1	98.8
Étang de Vaccarès	V_T2_P3	587.8	580.0	7.8	98.7
Étang de Vaccarès	V_T2_P4	387.8	151.3	236.5	39.0
Étang de Vaccarès	V_T2_P5	380.5	373.8	6.7	98.2
Étang de Vaccarès	V_T2_P6	1327.6	1293.5	34.1	97.4

The sample most landwards of the shoreline of the first Vaccarès transect and one from the Lironde contained the highest densities of plants from terrestrial habitats.

### 3.2. Correspondence analysis MAS

The correspondence analysis (CA) was done with 27 modern analogue samples, based on ecological groupings of aquatic and wetland species. When performing the correspondence analysis initially with all 30 samples, the inclusion of the three samples that were taken from dried out rivers or channels created too much noise, and these three samples were therefore excluded (although they are included in Table 1 and their positioning as supplementary variables in the CA was checked; the excluded samples from the Lironde (L_T2_P1/P2) grouped closely to the sample L_T5_P1, while the excluded sample from Saint-Saveur (L_T5_P2), which included only 3.6% of aquatic and wetland plants, grouped with samples V_T2_P1_1, V_T2_P3 and R_T2_P4).

**Fig 9:**
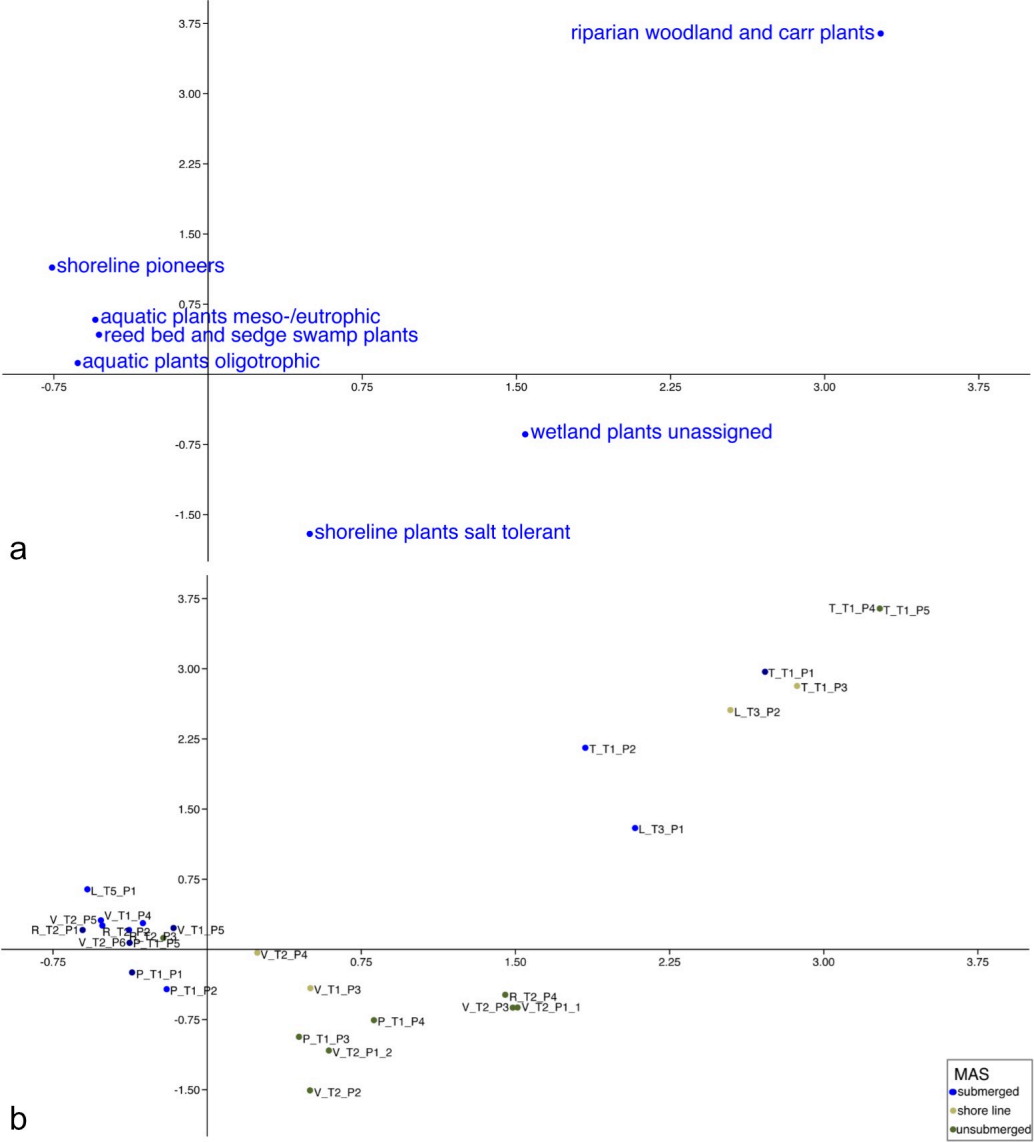
CA-graph based on density values of ecological groupings of aquatic and wetland plants in modern analogue samples. (a) Columns, (b) rows. Percentage of variation explained by the first two dimensions of CA: 58.1%.

The resulting biplot from the CA suggests three broad groupings amongst the sampled locations based on the density of ecological groupings (Fig 9A). Riparian woodland and carr plants positioned on the positive sides of both axes. Aquatic plants favouring oligotrophic as well as meso-/eutrophic conditions, reed bed and sedge swamp plants and shoreline pioneers positioned at the negative side of axis 1, but the positive side of axis 2. Salt tolerant shoreline plants as well as unassigned wetland plants positioned on the positive side of axis 1, but the negative side of axis 2. The positioning of the individual samples can be seen in Fig 9B. Samples from Tourtoulen (river Rhone) and the river Lez grouped on the positive sides of both axes with riparian woodland and carr plants. Samples from unsubmerged conditions were on the outer margin while samples from submerged conditions and from the shoreline spread more towards the middle. Samples from saline and brackish water conditions in the Camargue grouped in the other three parts of the graph. Most samples from unsubmerged conditions positioned in the positive side of axis 1, but the negative side of axis 2, with samples from the shoreline spreading more towards the middle. The only sample from unsubmerged conditions grouping with samples from submerged conditions was a sample from a reed belt from the Étang de la Grande Palun. The samples taken from submerged conditions grouped on the negative side of axis 1. The one sample from a canal near the Saint Sauveur area grouped here as well, despite the fact that its water was fresh during extraction of the sample.

When performing the CA based on individual species of aquatic and wetland species, the results were similar. The reed belt sample from the Étang de la Grande Palun was separated from the submerged samples and grouped with samples from the shoreline and samples from unsubmerged conditions. However, the sample from a canal near the Saint Sauveur area still grouped with submerged samples from saline and brackish water contexts.

### 3.3. General results of archaeological samples

The general results of the samples for the comparison with MAS have partly already been discussed by [88–91]. Therefore, mainly results regarding waterlogged remains of aquatic and wetland plants will be presented below (see S2 Table with the classification of the ecological groupings).

The samples contained on average 747.7 r/l of aquatic and wetland plants, but there was large variation between the samples as well. The richest sample (205038–1) contained 1821 r/l of aquatic and wetland plants, while the poorest sample (204097–1) contained only 3.3 r/l. In these samples, aquatic and wetland plants made up a much smaller proportion of the total, on average only 22.9%. The ecological groupings of aquatic plants from meso-/eutrophic conditions and unassigned wetland plants appeared in highest densities, while riparian woodland and carr plants as well as salt tolerant shoreline plants appeared in the lowest densities.

### 3.4. Correspondence analysis MAS and archaeological samples

**Fig 10:**
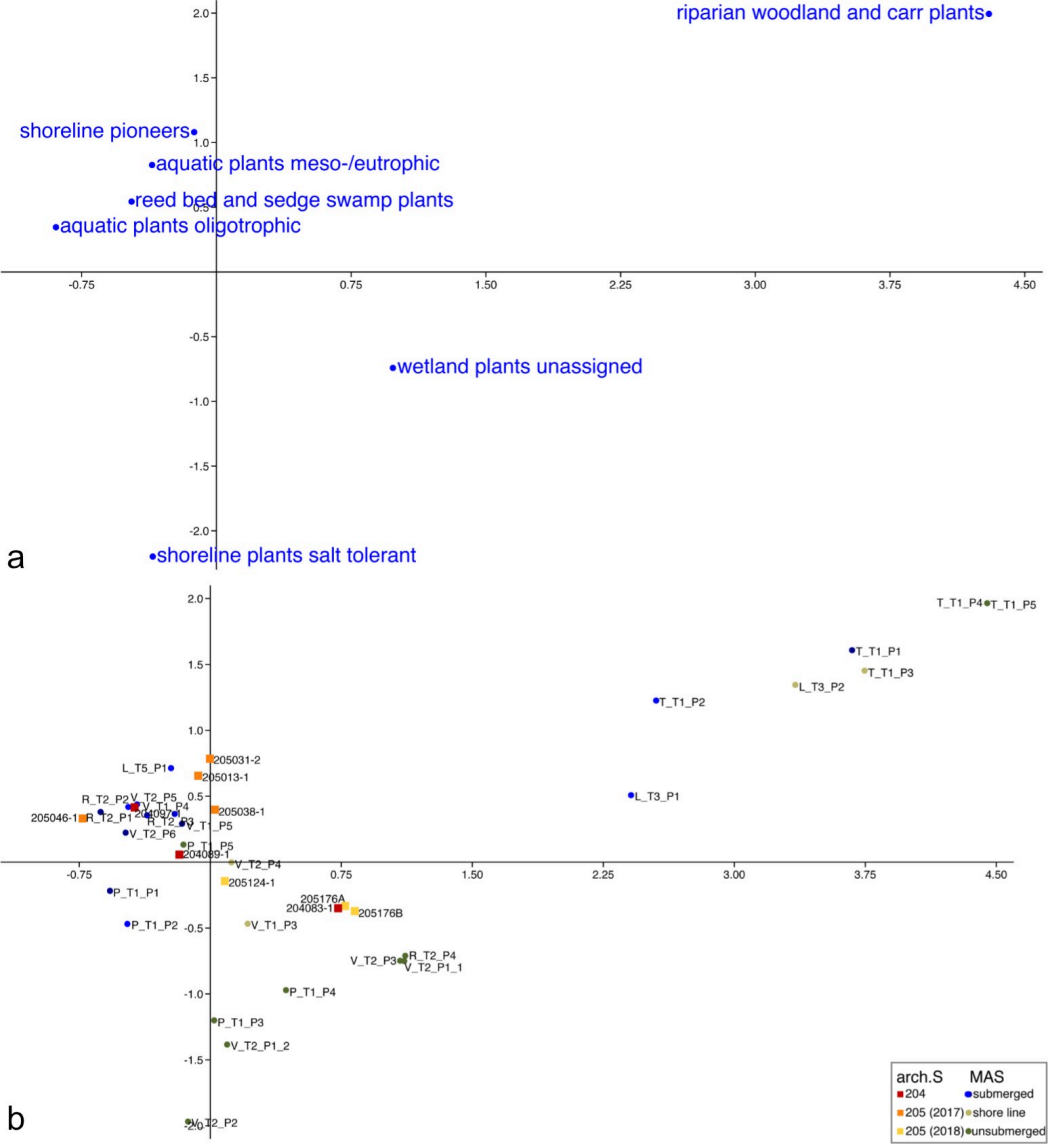
CA-graph based on density values of ecological groupings of aquatic and wetland plants in modern analogue and archaeological samples. (a) Columns, (b) rows. Percentage of variation explained by the first two dimensions of CA: 51.5%.

When including the archaeological samples in the correspondence analysis of the MAS, the distribution of the latter did not change much (Fig 10A). The distribution of the ecological groupings was similar to section 3.2, though the group of salt tolerant shoreline plants now positioned on the negative sides of both axes, further away from the group of unassigned wetland plants. The MAS were also still grouped the same way as in section 3.2. The archaeological samples from the zone 204 spread over modern analogue samples from submerged conditions (lowermost sample 204097–1) to MAS from the shoreline (sample in the middle 204089–1) to unsubmerged conditions (uppermost sample 204083–1; Fig 10B). The samples from zone 205 (2017) all grouped with MAS from submerged contexts. Three of those samples (205031–1, 205013–1, 205038–1) had a unique composition, setting them apart from the MAS and slightly towards the direction of the MAS from riparian contexts. The last and uppermost of the samples from this zone (205046–1) grouped with submerged samples at the other end of axis 2. The two samples from zone 205 (2018) from one side of the channel (205176A, 205176B) grouped with unsubmerged MAS while the sample from the other side (205124–1) grouped with MAS from the shoreline.

A CA using individual species was also carried out, but it was more difficult to interpret, certainly at least partly due to the invasive species in the MAS, mainly just causing the MAS to be separated from the archaeological samples on a majority of dimensions. The individual set of species of a sample superimposed most effects due to the different habitats.

## 4. Discussion

### 4.1 The separation of different wetland types in the MAS

The modern analogue samples were sorted into conclusive groups in the correspondence analysis, despite the fact that in some cases, diaspores could not be identified to the genus or species level.

Modern analogue samples from saline and brackish water lagoons could be well differentiated from samples of big fresh water rivers (Rhone, Lez, Fig 9). This separation was also present if individual species and not ecological groupings were used as a basis in the correspondence analysis. Although seed banks of saline water environments were not often directly compared to those of freshwater environments, there seem to be large differences between them, which could be one explanation for this clear separation. [113] found that seed banks play a much more important role in tidal fresh water marshes than in salt marshes, while [51] found differences in seed banks across the three main habitats forests, grasslands and wetlands. In a study on environmental variability of ostracods, [114] found that the proximity of fluvial channels influences the species compositions of these small crustaceans in different types of lagoon and marine environments.

Within the two big groups of saline and brackish water samples and fresh water samples (from big rivers), samples taken from submerged conditions (sublittoral) could also be separated from samples taken from unsubmerged conditions (eulittoral, supralittoral and epilittoral). As there were not so many fresh water samples (from big rivers), these results for this group have to be confirmed in further experiments, especially as samples from the shoreline could not really be differentiated from samples from submerged conditions. For the saline and brackish water samples, this separation of samples from submerged conditions of samples from unsubmerged conditions was clearer, as more samples were tested. Only one sample from unsubmerged conditions (P-T1-P5, Fig 9) grouped with samples from submerged conditions. This sample was taken from a reed belt far behind the shoreline (at the time of sampling) and contained many diaspores of reed bed and sedge swamp plants (notably *Schoenoplectus sp*. and *Phragmites australis*), which were responsible for this placement, as the sample contained no aquatic plants at all. If the correspondence analysis was done based on species instead of ecological groupings, this sample grouped ‘correctly’ with the other samples from unsubmerged contexts. This demonstrates why individual species should be tested as well if possible. However, it is also likely that this area is actually placed in submerged conditions at other times of the year. Samples from the shoreline grouped in between the submerged and the unsubmerged groups, as could be expected. As they were not very numerous, it should be tested in the future whether this separation of samples from the shoreline is really reliable. So far, studies of the relationships of water depth and emerging seedlings in experiments [115, 116] also confirm the relationship of water level and shoreline vegetation (reflected in the seed bank).

Within the samples from submerged conditions, no differences according to the depth of the sampling location could be seen. This could also be caused by the fact that samples were not taken in great depths, as the sampled lagoons were not very deep in general and the samples had to be taken at arm’s length (diving equipment was not available). It should be tested further whether submerged samples from greater depths can be separated, but differences of less than a metre cannot be distinguished based on the current data, which might also be caused by the fact that the tested waterbodies were all rather shallow.

The samples of small fresh water channels did not group with the fresh water samples (from big rivers), but rather with samples from saline and brackish water lagoons (L-T5-P1, Fig 9). The same applies to the two samples from the small fresh water river Lironde. However, the Lironde as well as one of the small fresh water channels were dried out during sample extraction and the results therefore might not be reliable, which is why those three samples were excluded from the final MAS database (they were tested as supplementary variables before exclusion). The comparative samples around Lattes could not be extracted at the same time of year as the ones from the Camargue, as they were not foreseen in the beginning of the project (with a duration of only one year). It remains to be tested whether these samples would have given better comparable results if they had been taken in the same season or at least during a time when the streams and channels were not dried out. It is essential to test more samples from smaller streams in the future in order to see whether they can be differentiated or not, and in the meantime, it is important to know that channels and smaller streams most likely cannot be differentiated in the current modern analogue dataset.

A more detailed identification of Amaranthaceae and Characeae could probably further improve the results, if the necessary reference material can be collected and compared in the future. Also, no replicate sampling was possible in this study, and it is therefore not clear how well one sample represents a habitat. It would be good to test this in studies as well.

The Rhone delta is fully embanked since the middle of the 19^th^ century with intensive water management and the presence of exotic species shows some influence of human activities on the vegetation. However, it is one of the most preserved coastal wetlands in France and the relationships between environmental factors and vegetation remains relevant in the context of the study. By taking MAS from this less anthropogenically influenced environment in combination with samples from the area where the archaeological samples originate from, we think we have created a solid data basis in order to interpret the archaeological samples of *Lattara*. Furthermore, this data could also be used to interpret various other coastal archaeological sites.

### 4.2 Wetland types represented in the archaeological samples

Many samples from the site of *Lattara* contained aquatic and/or wetland plants. These types of plants found their way into sediments of different zones, as was already shown in other articles about the site, e.g. [79, 81]. Of course, it cannot automatically be assumed that the wild plant remains ended up in the sediment by natural causes. It may be that the diaspores were charred or were found in special contexts (such as contents of vases), it is even highly unlikely. For this reason, only a small selection of samples coming from a similar context (a channel) was used for the comparison with MAS. Additionally, there were methodological differences in the treatment of the archaeological samples, like the use of different recovery techniques, which can possibly have an impact on the diaspore counts, see [94, 117, 118]. These methodological differences were not a main topic of this project, but they should be examined in more detail in the future. Nonetheless, the remains of aquatic and wetland plants have found their way into the sediment in some way and could therefore still give indications about conditions at or close to the site. It is assumed that the aquatic and wetland plants in the samples of the navigation channel ended up therein by unintentional local or regional transport [79, 119].

In comparison with the MAS, none of the archaeological samples directly grouped with fresh water samples from big rivers (Fig 10). This could have several reasons besides the absence of a direct influence of the Lez (or its two main branches) at the site.

Some of the taxa of riparian woodland are not normally found in archaeological sites because they are rather fragile (such as capsules of *Salix* and *Populus*, seeds of *Fraxinus angustifolius*) and some of the typical plant taxa from riparian woodland observed during the extraction of the MAS (e.g. *Rubus ulmifolius* in the *Rubus fruticosus* aggregate) could have been used as food plants at the archaeological site as well and were therefore not considered in the evaluation of archaeological samples. The problematic of robust diaspores of consumed plants with fruits containing many small seeds often found in samples from rivers should be looked at in more detail in the future (in MAS, diaspores of *Actinidia sp*. and *Ficus carica* also often appeared, compare to [34, 40]).

As mentioned in section 4.2, it is possible that samples from smaller fresh water streams or fresh water channels cannot be well separated, an additional factor as to why the influence of fresh water sources in the archaeological samples cannot be excluded based on the current database of MAS. Some samples from within the navigation channel did group towards MAS from riparian contexts and close to the sample from a fresh water channel near Saint-Sauveur. It is not yet entirely clear how the navigation channel was built and connected, although it is assumed that it was linked to the Lez oriental and the lagoon [54, p. 135]. Based on the comparison of archaeological samples from the navigation channel with modern analogue data, one possibility is that the water from the Lez was diluted by the lagoon and lost its typical characteristics of a big fresh water stream when entering the navigation channel (supposing that the preservation issues mentioned above did not play a decisive role). A study looking at pollen records of two neighbouring Languedoc lagoons found low amounts of pollen from fluvial sources (except *Alnus*, which is a high pollen producer), leading them to the assumption that fluvial sources had a minor influence on these lagoons [120]. The other possibility is that the navigation channel did not possess the same properties as a typical big fresh water river, but more like those of a smaller channel like the MAS tested in Lattes near the Saint-Sauveur area.

It seems likely that saline or brackish water sources also did have an influence on at least a part of the examined archaeological samples, which is also supported by the occasional finds of typical salt marsh plants like *Sarcocornia/Salicornia*, e.g. [79, 90], confirming the presence of this habitat in the archaeobotanical record after it was already predicted in other areas of the site by archaeozoological [82] and anthracological finds [86, 121]. The origin of these remains is yet to be determined. The comparison with MAS suggests that salt marshes were not overly prominent in the direct vicinity of the tested samples. Furthermore, in the CA including the archaeological samples, the ecological grouping of salt tolerant shoreline plants grouped further away from the ecological grouping of unassigned wetland plants, which could signify that there is a stronger differentiation between these groups in archaeological samples (most likely caused by the high amount of undetermined Amaranthaceae in MAS). It is therefore likely that more distant environments like the coastline or barrier beach are represented in the archaeological samples. It cannot be ruled out that *Sarcocornia/Salicornia* was deliberately brought to the site. In any case, the archaeological samples were not sedimented directly in a salt marsh environment.

### 4.3 Degree of water influence in the archaeological samples

In relation to the presence of water influence in the samples from *Lattara*, the investigated samples spread over the whole spectrum of MAS from submerged to unsubmerged conditions. Of course, this does not necessarily specify whether the archaeological sediments were primarily formed beneath water or not. It could also indicate the degree of water influence after an archaeological sediment was formed, but not sealed from further sedimentation. With this in mind, it can be said that some archaeological samples were strongly influenced by water, others less so. There was no clear pattern however, so the contexts of the individual samples have to be considered separately.

In samples from zone 204 [88, 89], the stratigraphically lowermost sample seemed to be the most strongly influenced by water, and that influence decreased towards the surface. This could have been linked to the filling of the navigation channel [54].

In the samples from zone 205 from the year 2017 [90], three samples grouped with submerged MAS, but were somewhat apart from the rest of the samples as they had a special composition with many shoreline pioneers, and grouped slightly more towards the riparian woodland MAS. The sampled spots of the navigation channel must have been covered by water during the sedimentation of all three of these samples during the process of filling up. The fourth and youngest sample of this set (205046–1) also grouped with submerged samples, but it had a completely different composition. Even though this sample contained less remains in general, high amounts of charred grains and lower overall densities of aquatic and wetland plants [90], it must also have been water-influenced at one point. By then, several centuries after the abandonment of the city, the navigation channel seems to have been abandoned and the area was used for other purposes (although it is still not clear for what other purposes; [55]). This change is also well-reflected in the composition of aquatic and wetland plants that grew at this location. It is possible that the amount of flowing water decreased towards this uppermost sample when compared to the MAS, resulting in a swampier environment than before, although this should be tested in further samples.

Two samples from the border of the navigation channel from zone 205 from the year 2018 grouped with unsubmerged MAS. They grouped very closely even though they do not belong to the same stratigraphic unit. The sample from the other side of the navigation channel grouped with MAS from the shoreline, indicating that the sampled spot on this side of the shore was closer to the water during the time that it was sedimented.

In this sense, it seems that each sample has to be examined individually. Depending on the position of the samples, they were more or less influenced by the water in the navigation channel during or after their formation. There might be a tendency of the samples to have slightly more characteristics towards riparian woodland earlier while the channel in zone 204 is gradually becoming clogged as the fresh water supply declined with time, which is supported by geomorphological findings.

As more samples containing waterlogged material will be analysed in the future, it will be possible to integrate them directly into this data set and thus defining the conditions around the navigation channel in more detail. At the same time, it will be possible to compare different sieving strategies used for the same sample to eliminate methodological differences.

It is also planned to look at the charred remains of aquatic and wetland plants found in association with crops in more detail using the comparison with MAS. This might help to define the conditions of cultivation of the crop plants used at the site.

## 5. Conclusion

In modern analogue samples from the Camargue and the area around Lattes, it was possible to distinguish samples of saline and brackish water environments from samples of (big river) fresh water environments. Within these two groups, it was further possible to separate samples taken from the sublittoral zone from samples taken from the eulittoral or further landwards of the shore. Samples from the shoreline grouped between those two categories. While it would be beneficial to expand the pool of modern analogue samples and look at methodological issues in more detail, these findings already provide a solid basis to improve the interpretation of taphonomic and formation processes of macrobotanical assemblages in archaeological samples of various sites in littoral lagoons and coastal areas based on less biased ecological data.

In samples from a navigation channel of the Gallo-Roman port city of *Lattara*, small-scale differences through space and time could be tracked by using the modern analogue dataset. The tested archaeological samples from the currently excavated navigation channel were sedimented under changing water levels in an environment that was not directly influenced by the Lez but might have resembled the smaller channels that can be found today in the area of the site. They were formed in a fresh water environment rather that a salt marsh. In accordance with geomorphological results, it was shown that the navigation channel clogged rapidly and lost its freshwater supply over a short period of time (approx. 100 years).

In the future, new samples from this site can be compared with the current dataset in order to get an idea about the type and degree of water influence and also to define palaeoecological characteristics of *Lattara*'s environment.

Samples from any other coastal archaeological site can easily be compared to the modern analogue dataset published with this article in an attempt to understand site formation processes in inhabited coastal areas in the past. Additionally, the dataset could be compared with other archaeological or natural samples across the world and could also be of interest for surveys and preservation of wetland habitats.

## Supporting information

S1 Table With densities of all plant remains in the modern analogue samples. Information about the samples can be found in the second sheet.
(XLSX)

S2 Table With densities of aquatic and wetland plant remains in the archaeological samples. Information about the processing oft he samples can be found in the second sheet.
(XLSX)
